# Within you, without you: HIV-1 Rev and RNA export

**DOI:** 10.1186/1742-4690-1-35

**Published:** 2004-10-29

**Authors:** Andrew I Dayton

**Affiliations:** 1 Center for Biologics Evaluation and Research, Food and Drug Administration, USA

## Abstract

Nucleo-cytoplasmic transport of RNA is one of many cellular pathways whose illumination has progressed hand in hand with understanding of retroviral mechanisms. A recent paper in *Cell *reports the involvement of an RNA helicase in the pathway by which HIV exports partially spliced and unspliced RNA out of the nucleus. This suggests the ubiquity of RNA helicases in RNA export from the nucleus, and has novel mechanistic implications.

## 

HIV must export to the cytoplasm not only full length genomic and structural gene-encoding RNA species, but also partially and fully spliced RNAs which code for a variety of overlapping structural and accessory genes. Whereas the fully spliced HIV RNA species readily exit the nucleus and undergo translation, the partially spliced and unspliced species require the interaction of the HIV-1 Rev protein with the cis-acting, viral RRE (Rev responsive element) for expression. Simpler retroviruses, such as the type-D Mason-Pfizer monkey virus (MPMV) solve a similar problem by encoding a cis-acting, constitutively active CTE (constitutive transporter element) in the 3'UTR of the viral genome, which uses a distinct pathway [1,2]. Of less clear significance is the existence of CRM-1 dependent nuclear export signals in gag proteins of HIV and RSV. It is theoretical possibility that shuttling gag proteins might contribute to nucleocytoplasmic export of genomic RNA, but it is equally possible that the only need for nuclear export of gag is to overcome the nuclear targeting signals late in viral replication [3,4]. Over the last seven years or so, tremendous progress has been made in characterizing nucleocytoplasmic transport pathways.

The Rev/RRE pathway involves the Ran-CRM-1, shuttling system: In the nucleus, Ran-GEF converts Ran-GDP to Ran-GTP. Ran-GTP bound CRM-1 binds the NES (nuclear export signal) domain of Rev, which is in turn bound to the RRE, and enables CRM-1 to transport the resulting RNA/protein complex as cargo into the cytoplasm, presumably through CRM-1 interactions with nucleoporins. In the cytoplasm, Ran-GAP converts Ran-GTP to Ran-GDP, releasing the REV/RNA cargo. The asymmetric distribution of Ran-GEF and Ran-GAP between nucleus and cytoplasm ensures a constant RAN-GTP/GDP gradient to facilitate CRM-1 recycling and continued Rev/RRE export. CTE-mediated RNA export is based on binding to the Tap-NXT complex, and export through an unrelated transport pathway, which, ironically, is responsible for the bulk of mRNA transport [2].

Enter the laboratories of Kuan-Teh Jeang and Larry Kleiman, who, in a comprehensive study in the October 29th issue of *Cell*, report the involvement of the RNA helicase, DDX3, in the Rev/RRE pathway [5]. Interestingly, DDX3 attracted their attention in preliminary screens indicating it was upregulated by the HIV-1 Tat protein, which is directly involved in transcriptional, not post-transcriptional regulation. Studying the effects of DDX3 overexpression, expression of dominant negative DDX3 mutants, or antisense knockdown of DDX3, variously on expression of Rev/RRE dependent reporter genes, Rev/RRE dependent subgenomic HIV constructs, or full length HIV genomes, Yedavalli et al. determined that in all cases, DDX3 had effects consistent with its playing a significant role in Rev/RRE dependent expression. These effects included expression of proteins, and nucleocytoplasmic partitioning of Rev/RRE dependent and Rev/RRE independent RNAs. In parallel studies, they determined that DDX3 had no effect on expression from CTE dependent constructs.

A number of findings not only provided evidence consistent with a role for DDX3 in the Rev export pathway, but also provided mechanistic insights. DDX3, though constitutively cytoplasmic, shuttles between nucleus and cytoplasm in a manner dependent on CRM-1, as evidenced by inhibition by leptomycin B (LMB), which inhibits CRM-1 export, but not Tap export. DDX3 also localizes to nuclear outer membranes in a speckle formation and co-immunoprecipitates with nucleoporins, in addition to having a distribution in the cytoplasm. In cell lysates, DDX3 co-immunoprecipitates with either Rev protein or CRM-1. Although the studies on cell lysates fail to distinguish between pairwise binding and ternary complex formation, ternary complex formation is a distinct possibility. DDX3 directly binds CRM-1 *in vitro *(in a manner independent of its NES). Although no data are presented concerning direct *in vitro *binding of DDX3 to Rev, for such an interaction to exist and to be significant, DDX3 would have to bind one of the three identified functional regions in Rev. These include the NES, the coincident NLS/RBD (nuclear localization signal/RNA binding domain) and the Rev multimerization domain, which flanks the RBD [6]. No regions in Rev besides these have been identified by mutagenesis or by any other techniques to be critical for function. Such an interaction would seem unlikely (though not impossible) in a complex of oligomerized Rev bound to RNA and CRM-1. Conceptually it might be easier to accommodate all the binding interactions in some kind of "pass-along" mechanism. This could allow some of the binding interactions to take place sequentially, rather than simultaneously.

In the tradition of good research, the results of Yedavalli et al. [5] pose as many questions as they answer. Though the binding interactions so far discovered, and DDX3's ability to shuttle between nucleus and cytoplasm could be consistent with a quarternary complex of Rev, RNA, CRM-1 and DDX3, the existence of such a complex remains to be proven (and the authors avoid implying its existence). It is possible there is some sort of "pass along" mechanism in which some of the binding interactions are sequential. It is tempting to suggest that DDX3 plays a role in relieving torsional tension to facilitate RNA passage through a confining nuclear pore, or that it helps remove certain proteins from RNA before, as, or after it reaches the cytoplasmic side of the nuclear membrane. Two other helicases have been implicated in RNA nuclear export pathways. In yeast, Dbp5p helicase, binds nucleoporins, localizes to nuclear pore complexes on the cytoplasmic side of the nuclear membrane, and is involved in mRNA export [7-9]. RNA helicase A (RHA) has been implicated in CTE-mediated transport [10-13]. A general requirement for RNA helicases in RNA transport is an attractive concept. Conceivably they could even be a significant molecular motor contributing to RNA transport: some RNA helicases are highly processive [see [14] and references therein] and may work by translocating along ssRNA, displacing bound RNA and protein (the "snowplow" model). At least one highly processive RNA helicase has been show to displace tightly bound protein from dsRNA [15]. An energy-dependent, highly processive RNA helicase tethered to the cytoplasmic side of the nucleopore could thus pull a long RNA molecule through the pore (see Figure 1). Theoretically this could solve the problem of how to get the rest of the RNA through the pore once the region initially targeted by CRM-1 (the RRE in the case of HIV) is transported and released. Other possibilities could, of course, include other molecular motors such as kinesin, in association with protein/RNA complexes [16].

**Figure 1:**
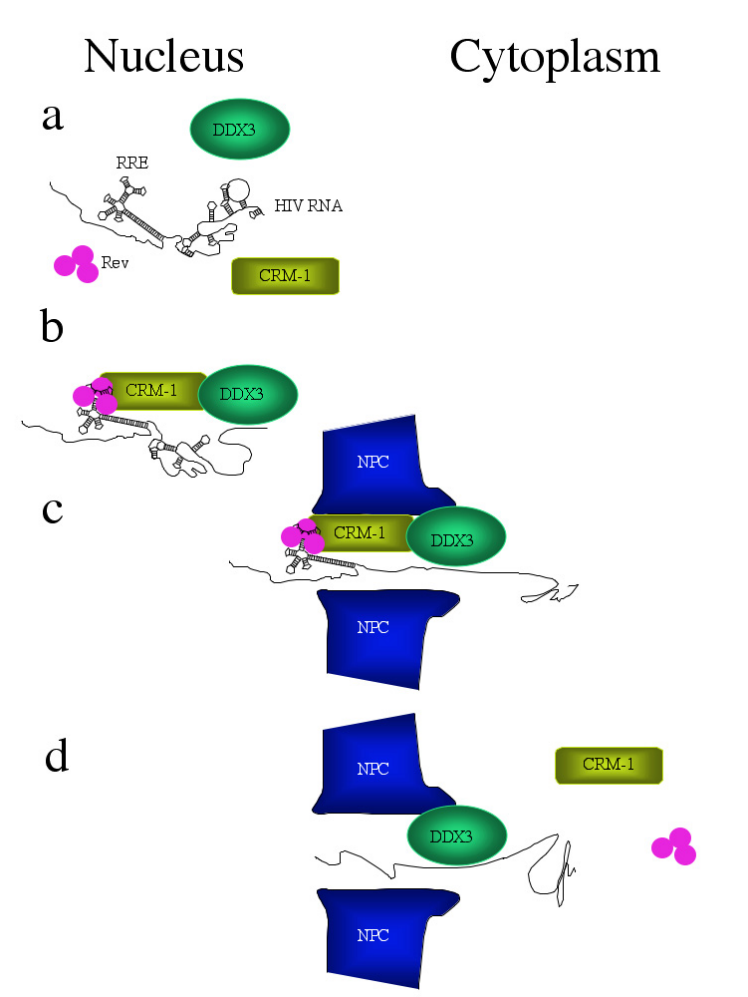
Schematic diagram outlining Rev mediated RNA export from nucleus to cytoplasm. **a**, Viral RNA develops secondary structure before binding critical protein components of the export pathway. **b**, Rev protein binds the RRE, forming a complex of REV, viral RNA, CRM-1 and DDX3, which begins to unwind secondary structure. **c**, The export complex enters the nucleopore, where both CRM-1 and DDX3 interact with nucleoporins. **d**, Even after CRM-1 and Rev are released from the export complex, DDX3 may still pull RNA through the complex by virtue of its processivity.

On a highly speculative note, the intimate involvement of a DEAD box helicase in the Rev pathway, and the observation that DDX3 has a cytoplasmic localization, in addition to its nuclear membrane localization, recalls some old data from two independent laboratories, indicating that in some systems Rev may not act on export, but on post export events, to promote Rev/RRE dependent expression [17,18]. The eIF4A family has been called the "godfather" of DEAD box helicases [19]. EIF4A3 can be targeted to the exon junction complex of spliced mRNA, and it has been speculated that it may have a splicing-dependent influence on mRNA translation [20]. In cell types where Rev does not act through export, could it act by specifically targeting to the ribosome a related DEAD box RNA helicase, translation initiation factor? Time will tell.

Finally there is the possibility that specific helicases will be valid therapeutic targets for antiretroviral chemotherapy. Although targeting a cellular gene involved in a viral pathway risks inhibiting a necessary cellular function, it avoids the overwhelming problem posed by rapid viral mutation to resistance.

## Abbreviations

RRE: Rev Responsive Element

MPMV: Mason-Pfizer monkey virus

CTE: Constitutive Transport Element

NES: Nuclear Export Signal

LMB: Leptomycin B

NLS: Nuclear Localization Signal

RBD: RNA Binding Domain

## Competing Interests

None. The opinions expressed are those of the author and do not necessarily express the opinion of the FDA.
